# Discussion of Dr. Taylor’s Paper

**Published:** 1907-07

**Authors:** 


### DISCUSSION OF DR. TAYLOR'S PAPER.

Dr. Dunbar Roy enjoyed the paper read by Dr. Taylor, and said that it was one of the best papers he had ever heard on the subject, as it was well handled and in a practical manner, and that he had covered the ground thoroughly. No matter what one's specialty is, headaches have to be contended with to some extent, and the danger lies in the fact that we are too likely to take a narrow view of the subject and consider everything caused by diseases included in our specialties. To avoid this general practice or hospital training is always an important preliminary before taking up a special line of work. Headache which appear in youths and gradually disappear as they grow older, has always interested him, and he asked Dr. Taylor for his opinion as to the probable cause of this variety.

Headache due to eye trouble, he believes, is overestimated by certain well-known eye specialists. As a rule, headaches occur in subjects with very slight errors of vision rather than in those with large degrees of error. Presbyopia has not been found as a frequent cause of headache. The individuals who have spasms of the ciliary muscles from overstraining are more likely to have it. The extrinsic muscles are not so often the origin of headaches as refractive errors. The time the headache usually comes on is important from a diagnostic standpoint. When they come on late in the day after excessive use of the eyes, as a rule it means they are due to a defect of vision. Dr. W. B. Armstrong mentioned the value of anointing the head with menthol dissolved in alcohol before placing the ice bag on it, as the results are then much more prompt and more marked than when the ice bag is used alone.

Dr. A. G. Hobbs did not think presbyopia ever caused headache. After 45 or 50 years of age, when presbyopia appears, there is usually some after cause to which the headache can be assigned. It is the borderline errors, rather than the severe ones, which are found to cause headache, and one is not infrequently in doubt as to whether to prescribe glasses or not. The age in which these are found is usually between twenty and thirty years. Never under 10.

Dr. H. M. Lokey said that many of the headaches that come on in the latter part of the night or the early morning are due to intranasal pressure from the gravitation of the serum into the diseased tissues as the patient lies on one side. Toxic absorption from over-eating or drinking the previous night is well-known as a cause of headache, but may be misleading.

Dr. C. P. Ward asked if Dr. Taylor thought that the prolonged use of certain patent medicines as "capudine" could cause, instead of relieve, a headache. He had one patient in whom he could find no other cause for a severe persistent headache.

He agreed with Dr. Armstrong that the benefit from the ice bag could be greatly augmented by using menthol and alcohol with it. Another simple and effective way to increase its value is by placing a rubber band around the head to press away the blood and thereby obtain more cold at the desired part. Intranasal pressure will cause a headache, but frequently it is due to interference with breathing, and the apparent proof of this is the readiness with which the pain will subside after the inhalation of oxygen.

Dr. Taylor (closing) said that he intended to have brought a sphynometer to demonstrate the method of taking the blood pressure. His idea as to presbyopia as a cause of headache was taken from a paper by a Philadelphia writer who otherwise gave a very clear exposition of the subject, and he thought it was reliable, but he would not vouch for nor attempt to defend his opinion. Some of the headaches of youths that disappear later are migraine, which he purposely omitted from his paper. Others may perhaps be due to the elasticity of the blood vessels which respond to slight causes, but as the person grows older they become less elastic and these insignificant causes are not enough to produce the headache.

The probable explanation of the reason that "capudine" relieved Dr. Ward's patient at first, but failed later, is that the origin of the headache has been progressing and developing, and finally reached a stage beyond which it could be relieved by ordinary mild sedatives.

